# Navigating the wild west: a review of guidance on clinical communications using personal BYOD, IM and third-party apps in the UK and Ireland

**DOI:** 10.3389/fdgth.2024.1457848

**Published:** 2025-01-06

**Authors:** Bernadette John, Ciara Heavin, Anthony Roberts

**Affiliations:** ^1^ Department of Restorative Dentistry, Cork University Dental School and Hospital, University College Cork, Cork, Ireland; ^2^ School of Business, College of Business and Law, University College Cork, Cork, Ireland

**Keywords:** IM and app use, policy adherence and training, General Data Protection Regulation (GDPR), communication best practice, patient data security, clinical decision support systems

## Abstract

**Introduction:**

The ubiquity of Bring Your Own Device (BYOD) personal smartphones, Instant Messaging (IM), and third-party apps, has made these technologies compelling for efficient communications between clinicians regarding patient care. However, the sensitivity of patient-related information necessitates secure, GDPR compliant modalities that prevent unauthorised access and ensure confidentiality. This scoping review explores existing guidelines, policies, and regulations that advise clinicians in the UK and Ireland on the secure use of these digital communication tools.

**Methods:**

Following the Joanna Briggs Institute (JBI) updated Framework for Scoping Reviews and the PRISMA ScR guidelines, this review examines the literature to identify relevant guidelines, policies, and regulations informing current clinical practice on the use of this technology. Academic databases including OneSearch, Embase, EBSCO, PubMed, Medline, and CINAHL were searched, in addition to hand searches of professional entities' websites, including trade unions, regulators, two national health systems, and several employers. Direct inquiries were made to 69 professional entities via telephone, email, websites, and X (formerly known as Twitter).

**Results:**

The review identified 18 papers that broadly recognise the importance of secure communication however, a lack of information on the appropriate selection or configuration of these popular technologies was evident. Most guidelines emphasise general security and data protection standards rather than providing clear actionable recommendations for technology use, thereby leaving a significant gap in technical guidance for clinicians.

**Discussion:**

There is a distinct lack of detailed, specific, consistent technical guidance available to clinicians. This review evidences an urgent requirement for enhanced guidelines that specify the most secure platforms, appropriate features, and configuration to maximise the security and confidentiality of clinical communications. Further research is recommended to develop comprehensive, actionable advice for clinicians.

## Introduction

The widespread adoption of smartphone technology has transformed global communication practices, with a significant uptake in the use of digital tools such as personal smartphones Instant Messaging (IM) and third-party apps (e.g., WhatsApp) for private communications between clinicians regarding patient care. These communications are well intended by clinicians as they strive for timely and optimal patient management, amidst stringent data processing requirements under the GDPR within the EU (1). In their publication on developing a core competency framework for clinical informatics professionals, Davies et al. (2) emphasise the critical need to enhance digital skills across the entire healthcare workforce to prepare for a digitally driven future where IT significantly influences clinical practice. This backdrop poses an essential question: *How do clinicians determine the most appropriate methods for communicating with each other using their personal devices?* Professional bodies—including trade unions, Royal Colleges, regulators, health systems [e.g., UK National Health Service (NHS) and Irish Health Service Executive (HSE)], and large and small private healthcare providers, provide clinical guidelines. These guidelines aim to inform clinicians on the full range of their professional duties, from clinical procedures to record keeping, confidentiality, consent, and communications.

A scoping review by John (3) evidenced growing international concern regarding the use of non-enterprise digital channels for confidential clinical communications among healthcare professionals noting that while practitioner reports revealed widespread adoption of apps and smartphones, they often lacked critical insights into the technical security measures required to safeguard patient data in compliance with GDPR (1). A recent survey revealed the widespread adoption of apps and smartphones for clinical communication between dental professionals in the Republic of Ireland and the UK, signifying a reliance on personal smartphone modalities for sharing clinical information, including images, often without fully understanding the data security implications of this practice (4).

In the USA, large financial institutions like Wells Fargo and BNP Paribas faced substantial fines in 2023 (114 million and 35 million euros, respectively) for their employees' unofficial use of messaging services such as WhatsApp for business purposes, thus avoiding regulatory oversight (5). Reflecting this trend, the UK's Information Commissioner's Office (ICO) reprimanded NHS Lanarkshire in 2023 when their staff were found to be improperly sharing patient data via WhatsApp, a method that violates existing policies (6). In a statement regarding the case, the Information Commissioner stressed the importance of securely handling sensitive patient data to maintain public trust in healthcare data management (6). The ICO emphasised that data protection standards must not be compromised. Although NHS Lanarkshire was not fined on this occasion, the incident highlighted the potential for significant penalties under the GDPR. Recent policy from the HSE (7, Page 4) explicitly classifies WhatsApp and IM as social media platforms and states; “*Data protection laws protect an employer where the employees’ use of social networking sites causes damage to that organisation's reputation or leads to the release of confidential information*”. This is a clear indication that the HSE interprets the liability for any breach lying with the employee, thereby highlighting the importance of clinicians adhering to policy and guidance on this issue.

In August 2022, an initial exploratory search of academic databases, to identify relevant guidelines available to clinicians across all 26 EU health systems yielded no results. Despite extensive efforts, including a thorough hand search of national health system websites and direct communication, the absence of publicly accessible policies was noted. The complexity of navigating these health systems websites, many of which were available in the English language but segmented by region or specialty, further hampered access to existing publications or policies. Prior searches, including inquiries to The European Observatory on Health Systems and Policies, the WHO's library, and the Wellcome Library, also proved fruitless.

This scoping review aims to identify and evaluate the existing guidelines, regulations, and policies available to clinicians in Ireland and the UK for managing sensitive communications between each other via personal BYOD smartphones, IM, and third-party apps. For the purposes of this study, “*clinicians*” refers broadly to healthcare professionals across various disciplines, including but not limited to doctors, nurses, dentists, and allied health professionals, who use personal digital devices to communicate with each other regarding patient care. The focus is specifically on scenarios where patient data is exchanged between professionals and the guidelines that inform these interactions.

## Methods

The methodology for this scoping review was informed by the Joanna Briggs Institute (JBI) Methodology (8) and adhered to the Preferred Reporting Items for Systematic Reviews and Meta-analysis guidelines for scoping reviews (PRISMA ScR). The JBI framework for scoping reviews was chosen due to its established rigour and suitability for mapping broad and complex evidence landscapes, and is particularly in areas lacking highly specific guidance, as is the case for clinicians' use of personal digital devices for secure communications. A scoping review approach was selected over a systematic review because it allows for a more comprehensive exploration of the types and scope of guidance available, rather than focusing solely on evaluating outcomes. Alternative frameworks, such as systematic review methodologies (e.g., PRISMA), were considered; however, these approaches did not align with the study's objective to map and synthesise a broad range of policy, guideline, and regulatory documents without evaluating specific intervention outcomes.

### Review question

What resources, (regulations, guidelines and or policies) are available to inform practice-based communications between clinicians using personal BYOD smartphones, IM and third-party Apps in the UK and Ireland?

### Eligibility criteria

#### Inclusion criteria

• Position statements, Regulatory documents, Guidelines or Policies issued by healthcare employers, health systems, regulators, or professional organisations including trade unions in the UK and Ireland.
• Targeted at clinicians, including but not limited to nurses, doctors, dentists, and health and social care professionals.
• Focused on secure, clinical communications between clinicians.
• Pertaining to the use of BYOD personal smartphones and or IM, third-party apps (such as WhatsApp, Signal or Siilo) platforms.
• Published in the English language post-May 2018, aligning with the implementation of General Data Protection Regulation (GDPR).

#### Exclusion criteria

• Publications not specifically concerning practice-based communication between clinicians using personal BYOD smartphones, IM, or third-party apps.
• Journal articles, including literature reviews, editorial or research papers.
• Publications that only concern social media sites for public engagement.
• Publications specific to the pandemic emergency period only.

Several significant publications by regulators such as the General Medical Council (9) the Medical Council of Ireland (10) and the Health and Care Professions Council (11) were excluded due to their lack of specific guidance regarding current technology, security considerations and obligations under the law.

### Sources

Given the challenges in identifying relevant publications during the preliminary searches, the focus was expanded to access policies and guidelines within the UK and Ireland via academic databases, hand searches of websites and by contacting relevant organisations directly via telephone and social media. The search was limited to guidelines, regulations, policy, and position statements by health systems, a broad range of regulators, professional bodies, public and private hospitals, and trade unions [such as the Dental Defence Union (DDU), Medical Defence Union (MDU), Irish Nurses and Midwives Organisation (INMO)] allowing for a comprehensive exploration of the guidance available.

### Search strategy

**Figure 1:**
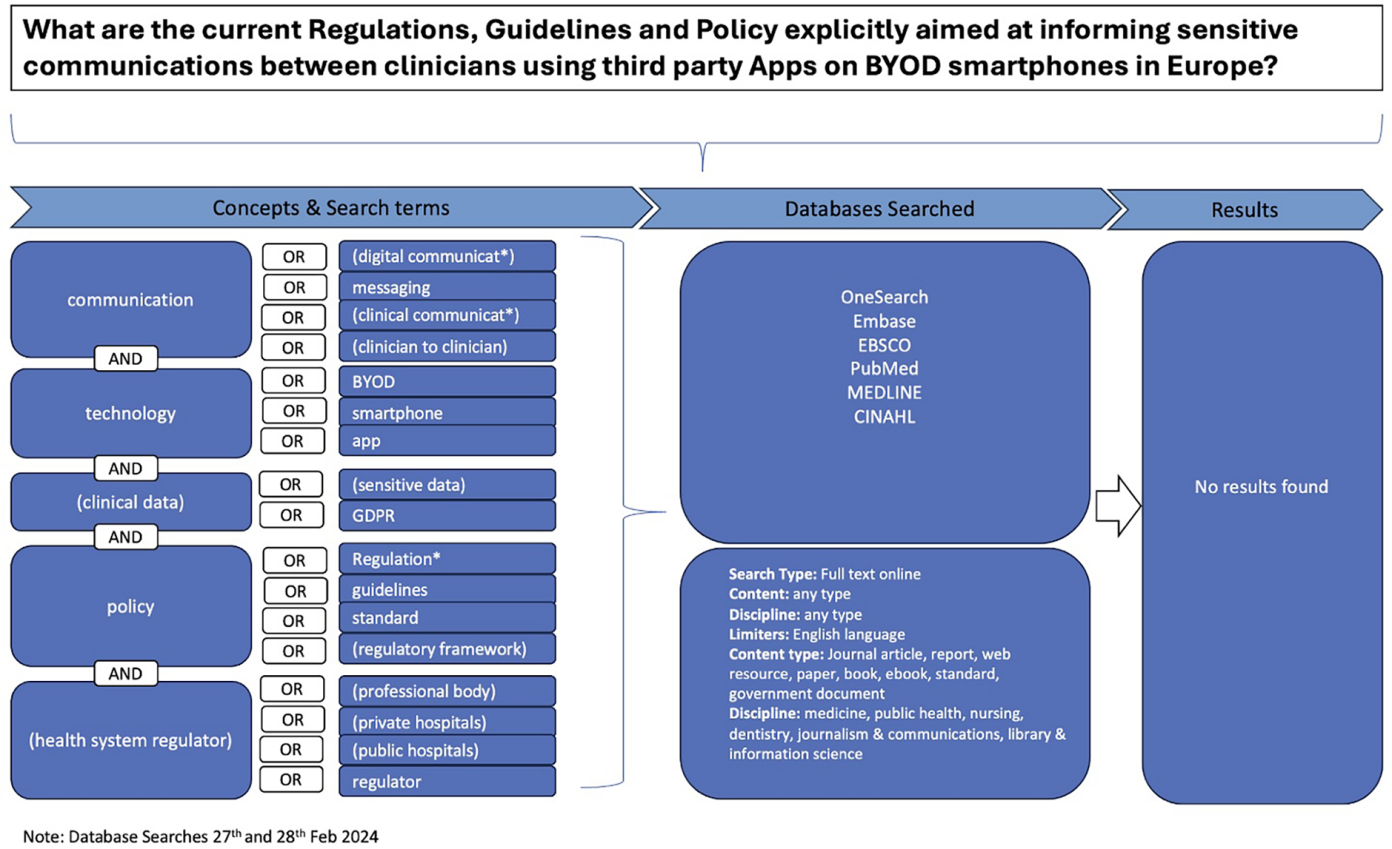
**Search Strategy Flow Diagram** This flow diagram outlines the comprehensive search strategy used to locate relevant publications informing current clinical practice. It details the steps taken to identify, screen, and select publications from electronic academic databases, general online searches, and direct inquiries to professional entities. The diagram visually represents the process of filtering publications based on inclusion and exclusion criteria, ultimately leading to the selection of (*n* = 18) publications for thematic analysis.

Despite a comprehensive search using keywords related to secure clinical communications via personal BYOD smartphones, IM, and third-party apps across academic databases including OneSearch, Embase, EBSCO, PubMed, Medline, and CINAHL no relevant results were identified (Figure 1). The paucity of guidance for clinicians in academic databases necessitated a broader approach that included hand searches of professional entities’ websites, direct inquiries via telephone, email, and social media to 69 organisations across the UK and Ireland (Supplementary Data Sheet 1).

### Study/source of evidence selection

Initial screening involved reviewing titles and abstracts against eligibility criteria, followed by full-text evaluation of potentially relevant publications. Data extraction was performed using a Data Extraction Form (DEF) to capture publication details, type of publication, geographical focus, and practical guidance on secure communications (Table 1). The draft data extraction tool was piloted, refined, and revised prior to the process.

**Table 1:** Snapshot of data extraction form (Def). Data Extraction Form (DEF) for Reviewing Publications. This table provides the variables extracted from the relevant publications during the review process. Key variables include citation details, publication year, publisher classification (e.g., regulator, health system, professional organisation), type of publication (e.g., regulation, guidelines, policy), the geographical focus of the publication, and whether the publication provides practical guidance for secure interprofessional communication using personal BYOD smartphones, IM, or third-party apps. The extracted data were systematically recorded to assist with the analysis and synthesis of the findings.

Included	Title	Author	Year	Author is Health System (HSE/NHS)	Author is Regulator	Author is Professional body	EU or Territory in which the paper is published	Inclusion criteria							Exclusion criteria			Rationale for inclusion
								Position statement, regulation, guideline, or policy issued by employer, health system, governing body, or professional organisation	Targeted at clinicians, including but not limited to nurses, doctors, dentists, & AHPs	Focused on/relevant to the topic of sensitive clinical communications between clinicians	Pertaining to the use of third-party apps (such as WhatsApp or Siilo) and/or instant messaging platforms	Addressing the utilization of smartphones/mobile devices and/or BYOD	English Language	Post GDPR, NOT specific to Pandemic	Not specifically concerning practice based communication between clinicians using via IM/3rd party apps, and personal mobile phones as BYOD	Journal articles, literature reviews, editorial or research papers	Concerning only social media for broadcast & public engagement	
Yes	Using Social Media	MDU	2024	NO	NO	YES	UK	YES	YES	YES	YES	NO	YES	YES	NO	NO	NO	This advice is the opposite to that of Dr Ellie Man from the same organisation in 2022, ’Smartphone safety’. Both pieces still available online.
Yes	Social media in the Republic of Ireland, top tips for doctors	MDU Ireland	2023	NO	NO	YES	Republic of Ireland	YES	YES	YES	YES	NO	YES	YES	NO	NO	NO	One paragraph only
Yes	HSE Social Media Staff Use Guidelines	HSE	2023	YES	NO	NO	Republic of Ireland	YES	YES	YES	YES	YES	YES	YES	NO	NO	NO	HSE “*REMEMBER: Data protection laws protect an employer where the employees’ use of social networking sites causes damage to that organisation's reputation, or leads to the release of confidential information*”. Mostly things are forbidden but that is good advice! Mentions “messaging apps”, Whatsapp and private messaging superficially
Yes	Bring Your Own Device (BYOD) guidance	NHS Trans Directorate	2023	YES	NO	NO	UK	YES	YES	YES	YES	YES	YES	YES	NO	NO	NO	Minimal advice for clinicians, better advice and links for IG professionals. It literally says you should be able to use your own device for work where there is no alternative, and your employer should ask you to sign a policy and what that policy should say.
Yes	Protecting patient data	DDU	2023	NO	NO	YES	UK	YES	YES	YES	NO	NO	YES	YES	NO	NO	NO	Really useful although appears to specifically avoid acknowleding communications and apps, the general advice it provides is useful. Concerns mobile phones and includes general advice on electronic data.
Yes	Health Records Management?	UHL NHS Trust	2023	YES	NO	NO	UK	YES	YES	YES	YES	YES	YES	YES	NO	NO	NO	This policy specifically includes text messages as health records.
Yes	Acceptable Use of ICT Policy	GOSH	2023	YES	NO	NO	UK	YES	YES	YES	YES	YES	YES	YES	NO	NO	NO	Relevant

### Data extraction

The initial screening of titles and abstracts was conducted by one reviewer assessing each against eligibility criteria. Potentially relevant publications were then retrieved for evaluation. The data extraction was systematically performed using the DEF, where citation details and all relevant key variables were recorded.

**Figure 2:**
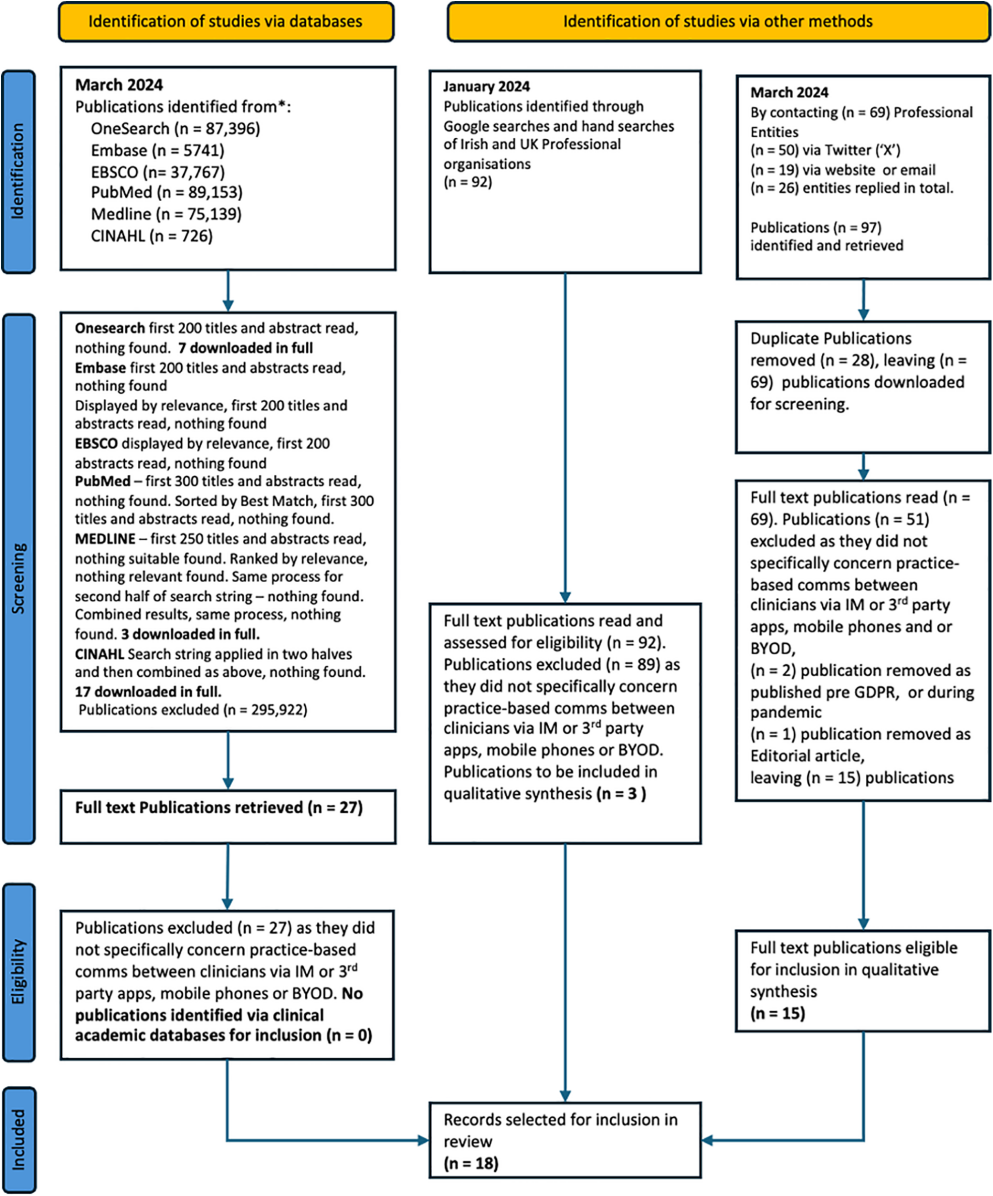
PRISMA ScR Flow Diagram. This flow diagram illustrates the Preferred Reporting Items for Systematic Reviews and Meta-analysis extensions for Scoping Reviews (PRISMA-ScR) process to identify, screen, and select the publications included in this review. It details each stage of the review process, including the initial identification of publications, the removal of duplicates, the screening of titles and abstracts, the assessment of full-text articles for eligibility, and the final inclusion of studies in the thematic analysis. The diagram provides a clear overview of how the review was conducted, ensuring transparency and reproducibility.

Discrepancies that arose during the screening and selection processes were resolved through discussion and consensus, involving all three reviewers. If necessary, authors were contacted to request additional data or clarify ambiguities. The inclusion and exclusion of publications, along with reasons for exclusion at the full-text stage, were meticulously documented following the PRISMA-ScR Guidelines to ensure transparency and reproducibility and are documented in the PRISMA-ScR flow diagram (Figure 2).

### Data analysis and presentation

**Figure 3:**
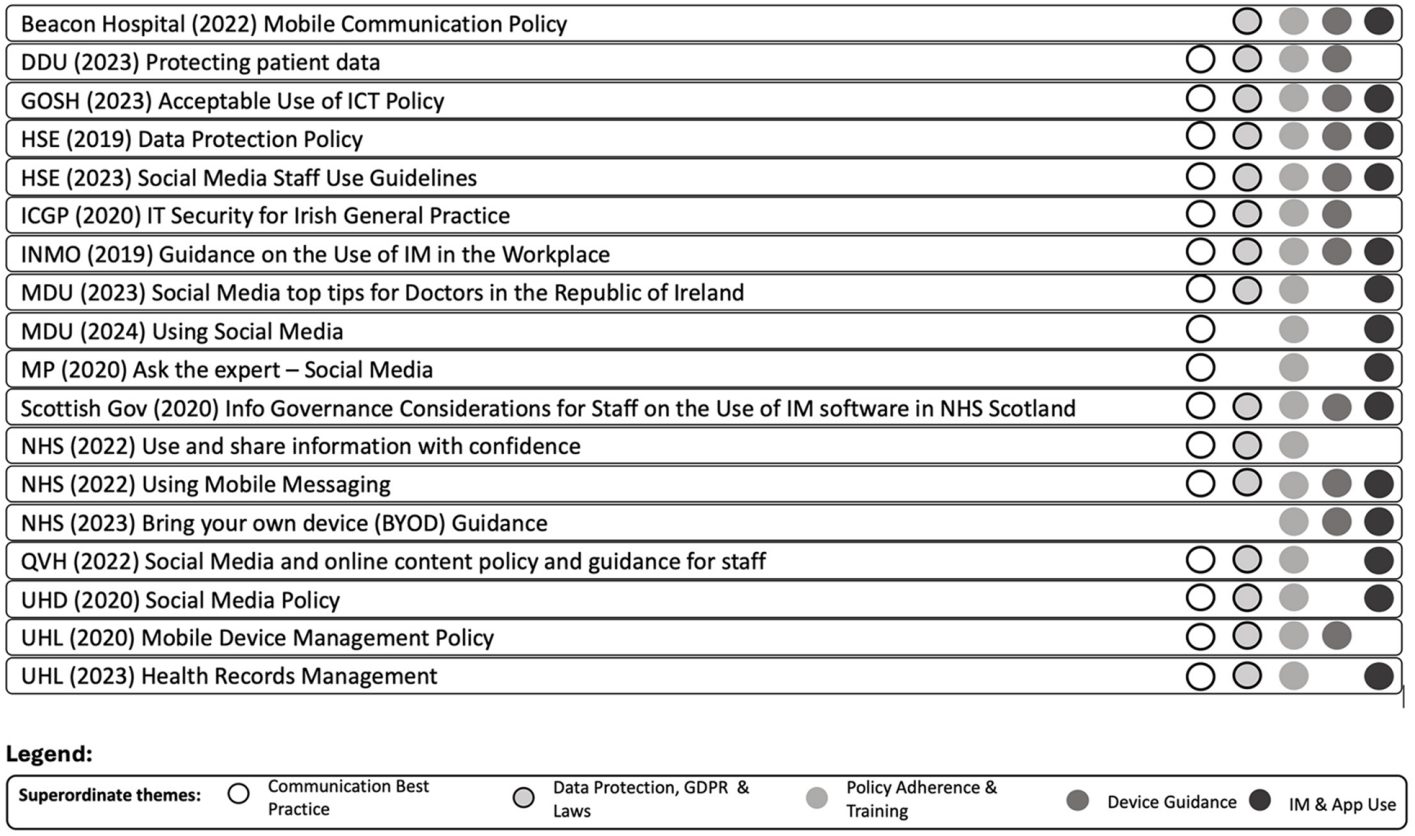
Publications mapped to superordinate themes. **Thematic Analysis of Publications** This figure provides a visual representation of the thematic analysis conducted on the included publications. It shows the five overarching superordinate themes and their associated subordinate themes, illustrating the interrelated elements of advice or data items identified within the publications. The figure highlights how the themes coexist and overlap, providing a comprehensive understanding of the guidance available for secure clinical communication using personal BYOD smartphones, IM, and third-party apps.

Data from the included studies were uploaded into EndNote citation management software and NVIVO a qualitative data analysis software to facilitate narrative synthesis and thematic analysis. In NVIVO, documents were systematically coded according to thematic categories based on Braun and Clarke's (12) six-step thematic analysis method. This method involved familiarisation with the data, generating initial codes, and reviewing these codes to identify recurring themes and common concepts across the publications (Figure 3). NVIVO's features, such as code categorisation, query functions, and visualisation tools, supported an organised approach to analysing patterns and consolidating insights.

## Results

The search strategy identified eighteen publications and Table 2 presents a visual overview of these publications alongside the five superordinate themes and eighteen subthemes developed from our analysis.

**Table 2:** Results - publications coded to superordinate themes and subthemes. Overview of Findings and Themes. This table presents a visual summary of the findings from the review, illustrating the relationship between the publications and the identified themes. It highlights the recurring themes and common concepts that emerged from the dataset, organised into overarching superordinate themes with associated subordinate themes. The table provides a structured overview of how the publications addressed issues such as confidentiality, consent, record keeping, data protection, and the use of personal devices and third-party apps for clinical communication.

Superordinate themes	Communication best practices			Data protection, GDPR & laws			Policy adherence and training				Device guidance					IM and App use;			No of codes	Total occurances
Subthemes	Confidentiality	Consent	Record Keeping	Access to data	Data Transfer	Rules and Regulations, Acts etc.	Cybersecurity Sources	Employer or Organisational Policies & Advice	Professional Guidelines, Codes, Policy etc.	Staff Training, Awareness & Support	BYOD Security	Smartphone Configuration	Legal/Regulatory Requirements	MDM Software	Network Security	Context Appropriate Use	Regulatory Scrutiny	App Selection		
Beacon Hospital (2022) Mobile Communication Policy	0	0	0	0	0	3	0	1	0	2	0	4	0	1	0	0	5	0	6	16
DDU (2023) Protecting patient data	3	0	1	0	0	4	0	4	4	0	2	2	2	0	0	0	0	0	8	22
GOSH (2023) Acceptable Use of ICT Policy	3	1	1	0	2	3	1	5	0	3	3	5	3	1	0	2	2	0	14	35
HSE (2019) Data Protection Policy	1	3	0	6	5	9	0	3	0	2	0	0	1	0	0	0	1	0	9	31
HSE (2023) Social Media Staff Use Guidelines	3	1	0	0	0	2	0	2	0	0	0	1	0	0	0	1	1	0	7	11
ICGP (2020) IT Security for Irish General Practice	1	1	1	0	1	0	0	0	2	0	0	6	1	0	1	0	0	0	8	14
INMO (2019) Guidance on the Use of IM in the Workplace	4	0	6	1	1	7	0	6	19	0	1	4	0	0	1	13	8	1	13	72
MDU (2023) Social Media top tips for Doctors in the Republic of Ireland	1	0	0	0	0	1	0	0	1	0	0	0	0	0	0	2	2	1	6	8
MDU (2024) Using Social Media	1	1	0	0	0	0	0	0	4	0	0	0	0	0	0	1	0	1	5	8
MP (2020) Ask the expert - Social Media	1	0	0	0	0	0	0	0	3	0	0	0	0	0	0	1	0	0	3	5
Scottish Gov (2020) Information Governance Considerations for Staff on the Use of IM software in the NHS Scotland	1	0	3	1	1	4	1	5	4	0	2	8	0	0	0	9	5	6	13	50
NHS (2022) Use and share information with confidence	1	0	0	0	1	1	0	2	0	0	0	0	0	0	0	0	0	0	4	5
NHS (2022) Using Mobile Messaging	2	0	1	0	0	1	0	10	1	0	2	7	0	0	0	22	1	4	10	51
NHS (2023) Bring your own device (BYOD) Guidance	0	0	0	0	0	0	0	1	0	0	1	0	0	0	0	0	2	0	3	4
QVH (2022) Social Media and online content policy and guidance for staff	1	2	1	0	0	1	0	1	1	0	0	0	0	0	0	19	5	0	8	31
UHD (2020) Social Media Policy	3	2	0	0	1	2	0	6	3	1	0	0	0	0	0	3	1	0	9	22
UHL (2020) Mobile Device Management Policy	2	0	2	0	0	1	1	10	0	6	13	16	1	7	3	0	0	0	11	62
UHL (2023) Health Records Management	1	0	11	3	1	8	0	12	4	2	0	0	0	0	0	1	0	0	9	43
Totals	16	7	9	4	8	14	3	14	11	6	7	9	5	3	3	11	11	5		
Key																				
Health Service Executive (HSE) Set																				
HSE (2019) Data Protection Policy																				
HSE (2023) Social Media Staff Use Guidelines																				
NHS Transformation Directorate Set																				
NHS Transformation Directorate (2022) Use and share information with confidence																				
NHS Transformation Directorate (2022) Using Mobile Messaging																				
NHS Transformation Directorate (2023) BYOD Guidance																				
University Hospital Leicester (UHL) Set																				
UHL (2020) MDM																				
UHL (2023) Health Records Management																				

### Communication best practice

A superordinate theme that emerged from the review in 16 of the publications is the provision of broad guidance regarding digital communications best practices, addressing the significance of confidentiality, consent, and record-keeping. This content was frequently relevant and coded to additional superordinate themes such as Data Protection, GDPR and Laws, Device Usage, IM, and Third-Party app Use, though they are present in varying degrees of detail.

#### Confidentiality

This subtheme was identified in 16 of the publications and broadly refers to key principles but offered little technical detail or practical guidance. Examples of such broad statements include “*individual dental professionals have an ethical duty of patient confidentiality, and must keep patient data from being mislaid or accidentally disclosed*” (13, Page 1).

#### Consent

Issues concerning consent were identified in seven publications. Gaining informed consent from patients for data sharing via IM or third-party apps is notably absent, with the focusing on staff adherence to BYOD policies and the broader implications of consent for public data sharing.

#### Record keeping

Record keeping issues were raised in nine of the publications, with guidance most often discussed in terms of principles, and scant acknowledgement of the technical and legislative requirements, standards or features required to ensure that records are kept securely, in compliance with the requirements of GDPR (1). For example, the DDU (13, Page 1) quotes directly from the GDC's standards (14, Principle 4.5) which states clinicians must “*keep patients’ information secure at all times, whether your records are held on paper or electronically*”.

### Data protection, GDPR and laws

This superordinate theme refers to guidance regarding data protection and compliance with laws and was raised in 15 of the papers. Few specific obligations were raised concerning how these laws apply to the use of personal BYOD, IM, or third-party apps for communications between clinicians.

#### Access to data

Four papers refer to a legal requirement for access to data in respect to rights such as portability or correction by data subjects (patients), or for personal data not to be transferred to third parties outside the European Economic Area (EEA) or for the protection of the data from destruction or damage, a requirement for with compliance with GDPR (1).

#### Data transfer

Eight publications superficially acknowledged the security requirements for the transfer of health-related data to comply with GDPR.

#### Rules, regulations and acts etc.

The importance of laws in general, from mentioning laws in general to signposting the specific requirements for clinicians process patient data using current “digital technologies” under particular acts or laws was found in 14 papers. However, their specific relevance to the use of BYOD smartphones, IM and third-party apps for secure communication was sometimes ambiguous.

### Policy adherence and training

This superordinate theme, found in all 18 papers, evidences where publications refer clinicians to additional resources such as professional codes of conduct, employer policies or dedicated cybersecurity sources, training, support, and awareness raising regarding the issues of concern.

#### Cybersecurity sources

Three publications signposted or asserted the value of specific, dedicated cybersecurity resources for further information. One publication referred clinicians usefully to the authoritative resources of the National Cyber Security Centre website (NCSC) (15) with two of the NHS Trusts referring to their own internal, dedicated *Cybersecurity* resources for any additional information (16, 17) specifically concerning the use of BYOD, IM and third-party apps for communications between clinicians.

#### Employer or organisational policies

The general importance of, or referral directly to specific additional relevant employer's or organisational policies is made in the 14 of publications for this subtheme.

#### Professional guidelines, codes etc.

The majority of publications (11) referenced additional professional guidelines, or codes of conduct. The resources referenced were often principle-based guidelines lacking specific detail on their integration into current personal communications technology with professional responsibilities described in general terms such as; “*the existence of IM platforms does not change the responsibility to maintain a comprehensive nursing/midwifery record*” (18, Page 6).

#### Staff training, awareness and support

This subtheme concerns not only training offered and the importance of awareness and understanding to ensure information governance compliance, mandatory training stipulated as a requirement, and signposting to relevant references and dedicated specialist resources. This was evidenced in six publications, all provided by large employers.

### Device guidance

This superordinate theme spanned the range of guidance offered to inform the use of BYOD personal smartphones for interprofessional communication and was offered across 10 papers.

#### BYOD security

Seven publications offer a range of guidance under this subtheme, from advising against the use of personal BYOD for clinical communications, to guidance for safer use, including non-technical but relevant “common sense” advice such as the importance of not allowing anyone else to access a device if it is used for confidential clinical communication. Signposting to the expert external resources of the NCSC for more detailed information “*specific to different operating systems*” is included in one document (15, Page 3).

#### Smartphone configuration

A range of advice regarding the configuration of personal smartphones was offered in this sub theme across nine publications. The Scottish Government (15) the INMO (18) and NHS (19) recommend disabling lock screen message notifications. Security suggestions vary, but include using secure passwords (13, 18) two-factor authentication (2FA) (19, 20), encryption, antivirus software (21), auto-lock and extra security settings. This sub theme also includes advice on the importance of remote wipe functionality and considerations for if a device is lost or stolen.

#### Legal/regulatory requirements

Five publications specifically address legal or regulatory requirements such as encryption as an important element of device security when transmitting or storing sensitive data. The security of any patient data [which is classified as 'special category' under GDPR (1)] streaming to a potentially insecure commercial cloud associated with a personal smartphone is mentioned in two publications (13, 21).

#### MDM software

Publications from three large employers (16, 17, 20) describe the value of the security features offered by Mobile Device Management (MDM).

#### Network security

Three publications mention network “*vulnerabilities*” or “*insecure networks*” with minimal detail on any action required to maximise security.

### IM and app use

This superordinate theme concerned a range of issues specifically concerning the use of IM and third-party apps, direct messaging on social media platforms across 14 of the publications.

#### Context appropriate use

A range of advice was offered across 11 of the publications regarding context appropriate use, from the importance of confirming that communications are sent to the correct person (15, 18, 19, 22) to the specific circumstances in which the use of such modalities would be acceptable, such as to alert a member of staff to a communication waiting for them on an approved enterprise channel (17), with a general emphasis on not sharing sensitive or confidential patient data via IM and third-party apps. Several publications condone the use of these tools to facilitate interprofessional communications where no sensitive personal data is shared (17, 22–24), in specific or emergency circumstances only (15), or where no alternative is available (19).

#### Regulatory scrutiny

The issues raised under this sub theme were mentioned in eleven publications. The statutory requirement for the availability of data/messages and images sent via IM or third-party apps, for access by Data Subjects (patients) in compliance with GDPR (25) (for correction and or portability and or deletion) and the availability of messages if requested for Subject Access Requests (SAR)s was described in five of the publications (15, 17–20). Several publications actively discourage (15, 18, 26) or completely forbid the use of IM and third party apps for the communication of patient information between clinicians (7, 17, 20, 22, 23) with assertions such as “*the current services on offer do not meet NHS information governance standards for the transmission of confidential information and their use for this purpose has been explicitly banned by NHS Digital*” (23, Page 9).

The requirement for any channels utilised for the processing of sensitive clinical data to be encrypted was mentioned in four publications (15, 17, 18, 27). One publication (20) points out that the conduct of a Data Protection Impact Assessment (DPIA) is mandatory where any sensitive information is being processed, which is relevant to the selection of an IM or third-party App (20).

#### App selection

Guidance in relation to app selection was provided in five publications. Information on how to evaluate an app's suitability for secure communication was provided in one publication (15), and information regarding specific configuration in order to maximise the security of sensitive data was provided in five publications (15, 18, 19, 26, 28).

## Discussion

### Ambiguity and inconsistency

This scoping review reveals a lack of consistent, detailed, actionable guidance regarding the protection of patient data when healthcare professionals use personal BYOD, IM, and third-party apps for communication between each other, across the majority of the publications. This ambiguity risks undermining the professional reputation of clinicians, both individually and collectively, and may attract regulatory scrutiny, sanctions from employers and substantial financial penalties for violations of GDPR (1).

The literature is unclear on digital communication best practices, despite the fact that health related data is categorised as a special *category*, requiring enhanced protection under GDPR (1). For example, few publications (15, 18, 22) advise clinicians to transcribe details and outcomes of clinical conversations conducted via personal BYOD, third-party app and IM into patient records and all leave the question of whether it might be more effective to directly download these exchanges as PDFs and integrate them into electronic health records (EHR) unresolved. This process would provide a reliable audit trail, save time, and meet the requirements of Freedom of Information or Subject Access Requests (15, 18, 20, 29). Furthermore, if deletion of the data is necessary, the importance of removing it from both the sender's and all recipients' devices (19) should also be addressed consistently across publications. Clinical images constitute medical records and must be preserved (30) so this area of ambiguity must also be addressed, and any inconsistencies resolved.

Several publications describe a requirement for data minimisation of confidential communication information via personal smartphones, third-party apps or IM (15, 16, 19, 22, 24). However, the INMO (18) alone acknowledges the risks of confusion regarding patient identity, a concern established by scholars in the literature (31, 32) and considered “*crucial for safe healthcare delivery*” (32, Page 8).

As previously noted, the HSE (7) classifies WhatsApp and IM as social media, advising against sharing confidential information via such modalities, yet proactive policy communication is unclear. Several publications actively discourage (15, 18, 26) while others completely forbid the use of personal BYOD, IM and third-party apps for the communication of patient information between clinicians (7, 20, 22, 23). with assertions such as “*the current services on offer do not meet NHS information governance standards for the transmission of confidential information and their use for this purpose has been explicitly banned by NHS Digital*” (23, Page 10). Significant variations in the guidance offered exist across all publications and this can cause confusion.

### Adherence to policies and training

There is a potential flaw in how existing policies are promoted and how the importance of regular training is emphasised. While some public health systems and large employers have dedicated cybersecurity resources, including specialist staff and technical support, there is a requirement for comprehensive, consistent policies and training programs addressing the specific concerns regarding the use of current BYOD personal smartphones IM, third party apps for secure interprofessional communications in clinical settings. Coordination between employers, unions and regulators could enhance training for all clinicians, including those in specialist training (who can be mobile between employers nationally with differing policies) and those employed in small private practices, who may not even be aware of the availability of MDM software or their own statutory obligations under GDPR (1).

A raised awareness on the importance of regular compulsory training on the relevant areas (cybersecurity, the law etc) could become part of annual Continuous Professional Development (CPD) enforced by the regulators. The fact that the Government of Scotland provided some of the most detailed, comprehensive, and specific guidelines we accessed (15), flags issues regarding how well this guidance is communicated, in order to have meaningful impact on clinical practice, as evidenced from the recent ICO sanction of NHS Lanarkshire in 2023 (6).

The comprehensive range of support and mandatory education and training around cyber security and information governance described in several of the large employer policies (16, 17, 20, 23, 29, 30) would be valuable for all clinicians, especially for those who do not work for large employers, without dedicated support.

### Technical guidance for secure communication

There is a gap in the provision of detailed considerations, technical requirements, and guidance regarding current popular communication modalities in healthcare. Few publications offer well informed, practical advice or signpost expert resources to inform the safer integration of these digital tools into clinical practice while maintaining data security. The literature focuses on general principles rather than providing detailed statutory requirements or specific technical guidance. Most publications offer high level recommendations on data security practices without including concrete technical details, such as configuration for apps or devices (7, 13, 15–19, 21–24, 26, 28–30, 33). This hampers clinicians' understanding of data protection obligations and requirements, particularly for GDPR compliance where even an IP address is considered personal data (23, Page 5). Clinical images with EXIF data (including geographical coordinates, date, time, and device details) (34) are also personal data, yet this is not addressed in any of the reviewed publications. Only two publications highlight the requirement for DPIA when processing sensitive information (20, 29), indicating a widespread lack of awareness of essential data protection procedures. The failure to reference specific security requirements for patient health data using BYOD, IM, or third-party apps suggests a lack of awareness among clinicians (24, 28).

This review highlights significant gaps in guidance provision in terms of actionable, specific recommendations. The existing literature and policy guidance vary widely, with some sources emphasising general principles and others offering more detailed but inconsistent advice. This disparity highlights the need for harmonisation of well-informed guidance to ensure clinicians have access to both practical advice and regulatory clarity. Comprehensive, technically detailed guidelines are needed, especially for clinicians in private practice without specialist IT or cybersecurity support. A unified approach, directing healthcare professionals to authoritative resources such as the NCSC (35–39), could enhance decision making and patient data security practices.

## Limitations

The restricted access to documentation, available only to full members with paid memberships, hampered our ability to gather a comprehensive set of publications for analysis. This limitation may have affected the generalisability or accuracy of our findings but also highlights a significant gap in accessibility.

## Conclusions

This scoping review highlights the complex landscape of guidelines and policies for personal BYODs, third-party apps, and IM for interprofessional clinical communication in the UK and Ireland. The absence of appropriate guidelines poses challenges for clinicians, particularly those outside large healthcare employers, who may lack access to specialised resources, dedicated technical support, policy, and training, exacerbating any disparities in information security and data protection practices. Existing guidance is often superficial, inconsistent, and does not appear to be informed by up-to-date expertise, threatening patient confidentiality and data security. There is an urgent requirement for harmonised, contemporary guidelines that can adapt to evolving technology.

The specialist discipline of health informatics has emerged to address the requirement for uniquely skilled professionals to design, develop, implement, and evaluate health information technology, which highlights the extensive body of evidence and tools existing in this field (40). Drawing upon established work from Healthcare, Health Informatics, and areas such as Information Systems (IS) is essential to inform the research and development of a comprehensive, holistic model of data quality dimensions to assess the quality of data/information currently informing clinical practice in this area. Such a model is essential for identifying and addressing gaps in current guidance. A collaborative approach involving healthcare providers, regulatory bodies, trade unions, cybersecurity experts, legal advisors, IS and health informatics specialists is vital for developing and disseminating guidance that keeps pace with technological advances, ensuring the highest standards of data protection, patient confidentiality, and professionalism in clinical communications.

## Data Availability

The original contributions presented in the study are included in the article/Supplementary Material, further inquiries can be directed to the corresponding author.
