# Supplementary material for: Navigating the wild west: a review of guidance on clinical communications using personal BYOD, IM and third-party apps in the UK and Ireland
Source: Front Digit Health. 2025 Jan 6;6:1457848. doi: 10.3389/fdgth.2024.1457848 (PMC11743480; doi:10.3389/fdgth.2024.1457848)
Supplement: Supplementary file 1 [file Datasheet1.pdf]

Entities Contacted

Number	Name	"X" Handle	Contact Details	Country
1	Royal College of Anaesthetists	@RCoAnews		UK
2	British Dental Association (BDA)		enquiries@bda.org	UK
3	Faculty of Dental Surgery at The Royal College of Surgeons	@FDS_RCS		UK
4	Faculty of Dental surgery of Edinburgh (Royal College of Surgeons of Edinburgh)	@RCSEdFDS		UK
5	Royal College of Emergency Medicine	@RCollEM	communications@rcem.ac.uk	UK
6	Royal College of General Practitioners	@RCGP		UK
7	Faculty of Intensive Care Medicine	@FICMNews		UK
8	Royal College of Obstetricians and Gynaecologists	@RCObsGyn		UK
9	Faculty of Occupational Medicine	@FOMNews		UK
10	Royal college of Ophthalmologists	@RCOPhth		UK
11	Royal College of Paediatrics and Child Health	@RCPCH		UK
12	Royal College of Pathologists	@RCPATH	comms@rcpath.org	UK
13	Faculty of Pharmaceutical Medicine	@FacultyPharmMed		UK
14	Royal College of Physicians of Edinburgh	@RCPEdin		UK
15	Royal College of Physicians of Ireland	@RCPI_news		Ireland
16	Royal College of Physicians	@RCPhysicians		UK
17	Royal College of Physicians and Surgeons of Glasgow	@rcpsglasgow		UK
18	Royal College of Psychiatrists	@rcpsych	press@rcpsych.ac.uk	UK
19	Royal College of Psychiatrists in Scotland		pressscotland@rcpsych.ac.uk	UK
20	Faculty of Public Health	@FPH	JulianRyder@fph.org.uk<br>news@fph.org.uk	UK
21	Royal College of Radiologists	@RCRadiologists		UK
22	Faculty of Sexual and Reproductive Health	@FSRH_UK		UK

23	Royal College of Surgeons of Edinburgh	@RCSEd	pressoffice@rcsed.ac.uk<br>rcsed@tigerbond.com<br>comms@rcsed.ac.uk	UK
24	Royal College of Surgeons of England	@RCSnews	PressOffice@rcseng.ac.uk<br>(+44) 20 7869 6053	UK
25	Royal college of Surgeons of Ireland	@RCSI_Irl		Ireland
26	Faculty of Sport and Exercise Medicine UK	@FSEM_UK		UK
27	Royal College of Nursing	@RCN	Contact Box on RCN Website	UK
28	Royal College of Midwives	@MidwivesRCM	Contact Box on RCM Website	UK
29	Nursing and Midwifery Board of Ireland	@NMBI	EducationandGuidance@NMBI.ie	Ireland
30	Irish Nurses and Midwives Organisation (INMO)		aileen.garrihy@inmo.ie	Ireland
31	Health Service Executive (HSE)	@HSELive	Kavish.Naicker@hse.ie	Ireland
32	General Medical Council (GMC)	@GMCUK	gmcpress@gmc-uk.org<br>standards@gmc-uk.org<br>(+44) 161 923 6602	UK
33	Irish Medical Council	@MedCouncilIRL	educationandtraining@mcirl.ie<br>rachael.kelly@mcirl.ie	Ireland
34	College of Anaesthesiologists of Ireland	@COAIrl		Ireland
35	MediSec (profesional indemnity)	@MedisecIreland	info@medisec.ie	Ireland
36	Irish College of General Practitioners	@ICGPnews	library@icgp.ie	Ireland
37	Irish College of Opthalmologists	@eyedoctorsIRL<br>D39@IALabs<br>@Vision_Irl		Ireland
38	The Irish Dental Association (IDA)	@IrishDentists		Ireland
39	Dental Council of Ireland	@DentalCouncilPr	Ino@dentalcouncil.ie	Ireland
40	The British Association of Oral Surgeons (BAOS)	@BAOS_UK		UK
41	The British Orthodontic Society (BOS)	@BOSbraces		UK
42	Irish Society for Dentistry for Children	@isdc_ie		Ireland

43	Orthodontic Society of Ireland	@Orthosocirl- OSI		Ireland
44	Queen Victoria hospital trust		qvh.communications@nhs.net	UK
45	The Medical Defence Union (MDU)	@the_mdu		UK
46	The British Medical Association (BMA)		SAdmin@bma.org.uk<br>support@bma.org.uk<br>SAdmin@bma.org.uk<br>(+44) 300 123 1233	UK
47	Princess Royal University Hospital (PRUH), CEO	@AngelaHelleur		UK
48	Midwife for the Highlands and Islands, Confidential Cpersonal Communication asking for her NHS Trust policy			UK
49	The Nursing and Midwifery Council (NMC)	@NMCnews	media@nmc-uk.org	UK
50	St Vincents hospital		Dataprotection@st-vincents.ie<br>dataprotection@st-vincents.ie	Ireland
51	Great Ormond St Hospital (GOSH)		SAR@gosh.nhs.uk<br>media@gosh.nhs.uk	UK
52	General Dental Council (GDC)	@GDC_UK	communications@gdc-uk<br>policyinbox@gdc-uk.org	UK
53	Medical Protection (MP)	@MPS_Medical	education@medicalprotection.org<br>replyeventsupport@medicalprotection.org	UK
54	The Mater Private Hospital	@materprivate	dataprotectionofficer@materprivate.ie	Ireland
55	Blackrock Health (Private Health Group)		dpo@blackrockhealth.ie	Ireland
56	Blackrock Clinic (Private Hospital)		DPO@Blackrock-clinic.com	Ireland
57	Hermitage Clinic (Private Hospital)		dpo@hermitageclinic.ie	Ireland
58	Galway Clinic (Private Hospital)		dpo@galwayclinic.com	Ireland
59	The Beacon Hospital (Private Hospital)		dataprotection@beaconhospital.ie<br>margaret.callanan@beaconhospital.ie	Ireland
60	St James Hospital		dataprotection@stjames.ie	Ireland

61	National Health Service (NHS)	@NHSuk		UK
62	Cork University Hospital (CUH)	@CUH_Cork		Ireland
63	The Dental Defence Union (DDU)	@the_ddu		Ireland
64	University Hospital Dorset NHS Trust (UHD)		richard.moremon@uhd.nhs.uk	UK
65	Kings College Hospital NHS Trust		kch-tr.FOI@nhs.net<br>https://www.kch.nhs.uk/document/freedom-of-information-request-form	UK
66	Leicestershire Partnership NHS Trust		https://www.leicspart.nhs.uk/about/freedom-of-information-requests/ website	UK
67	The Mater (HSE Public Hospital)		info@mater.ie<br>(+353) 1 803 2333	Ireland
68	Leicestershire Partnership NHS Trust		LPT.DataPrivacy@nhs.net	Ireland
69	The Wellcome Trust		https://wellcome.org/who-we-are/contact-us	UK
